# Intestinal Schistosomiasis as Unusual Aetiology for Acute Appendicitis, Nowadays a Rising Disease in Western Countries

**DOI:** 10.1155/2012/896820

**Published:** 2012-06-26

**Authors:** I. López de Cenarruzabeitia, S. Landolfi, M. Armengol Carrasco

**Affiliations:** ^1^General Surgery Department, Clinic University Hospital of Valladolid, Level 3, 47011 Valladolid, Spain; ^2^Histology Department, Vall d'Hebron University Hospital, Histology Department Building, Passeig de la Vall d'Hebron, 119-129, 08035 Barcelona, Spain; ^3^General Surgery Department, Vall d'Hebron University Hospital, Level 4, Passeig de la Vall d'Hebron, 119-129, 08035 Barcelona, Spain

## Abstract

Intestinal schistosomiasis as unusual aetiology for acute appendicitis, nowadays a rising disease in western countries. Recent changes in global migration has led to an immigration growth in our scenario, upsurging people coming from endemic areas of schistosomiasis. Schistosomal appendicitis, seldom reported in developed countries, is now an expected incrising entity in our hospitals during the near future. Due to this circumstances, we believe that schistosomiasis should be consider as a rising source for acute appendicitis in western countries. In order to illustrate this point, we present a case of a 45-years-old black man, from Africa, was admitted via A&E because of acute abdominal pain, located in right lower quadrant. Acute appendicitis was suspected, and he underwent laparotomy and appendectomy. Pathological study by microscope revealed a gangrenous appendix with abscesses and parasitic ova into the submucosal layer of the appendix, suggesting Schistosomiasis.

Schistosomal appendicitis is rarely reported in developed countries. However, recent changes in global migration have led to an immigration growth, from endemic areas of schistosomiasis, in Western countries. These changes are building a new scenario in which an ascending number of schistosomiasis is expected in our hospitals in the near future. Due to this circumstances, we believe that schistosomiasis was unusual in the past in our environment, nowadays it should be considered in our landscape as a familiar cause for different medical entities, as in our case, for acute appendicitis.

Schistosomiasis is the second most prevalent parasitic disease worldwide. More than 200 million people are infected, 120 million are symptomatic, and 20 million suffer from severe disease. An estimated 85% of all cases, and virtually all of the most severe, are concentratedin African countries. Brazil, China, and Yemen are the most affected countries in the Americas, Asia, and the Middle East. Population growth and movement in endemic areas and ecologic changes resulting from increasing use of water for irrigation and electricity generation have contributed to the spread of infection [1]. Bierman and colleagues state that there is a steady rise of imported schistosomiasis cases in industrialized countries, which may reflect both a true increase and raised awareness [2]. TropNetEurope, a group of tropical medicine institutions in Europe, reported more than 800 cases in 2003, mostly from sub-Saharan Africa (*T. Jelinek, personal communication, April 2004*). Two-thirds concerned immigrants and refugees, and only every seventh case involved is a European tourist. The same group raise awareness in relation to imported schistosomiasis in Europe analysing epidemiologic and clinical features of 333 patients with schistosomiasis, in this study Schistosoma Mansoni has been involved in most cases [3]. No formal national or international statistics exist because schistosomiasis is not a notifiable disease. High exposure among travellers occurs during adventure and diving tours to African destinations such as Malawi and Victoria lakes, the Dogon country, and the Omo River [4].

Schistosomiasis, also known as bilharzia, bilharziosis, or snail fever, is a parasitic disease cause by a trematoda (Platyhelminthes or flat worms) of the genus *Schistosoma* (*S. Mansoni*, *S. Japonicum*, *S. Haematobium,* etc.). It is endemic in regions of Asia, Africa, and South America, especially in areas with water that is contaminated with infected freshwater snails, which may carry the parasite. Due to global migration changes, the disease is increasingly affecting developed countries.

Although it has a low mortality rate, schistosomiasis is often a chronic illness that can cause liver and intestinal damage. In some chronically developed cases the patient can die secondary to its complications. The relevance of an early diagnose is important, based on two features, in one hand could prevent disease progression at individual and social level, and on the other, its treatment with an anti-helminth drug (Praziquantel) orally prescribed is very efficient, easy to use and with prompt effect.

Schistosomiasis as accountable for acute appendicitis are reported between 0.02%–6.3%, representing 28.6% of Chronic appendicitis in endemic areas [5]. Reports coming from industrialized countries, as Japan, reviewing 311 specimens founded an incident of 0.32% [6].

A 45-years-old black man, from Africa (Sélibaby), was admitted via A&E because of acute abdominal pain located in right lower quadrant concurrent with 4 days of high temperature (38°C). As clinical history, he had had double-heart valve replacement (Aortic and Mitral valve) 3 months ago in another hospital, currently on Acenocoumarol as anticoagulant therapy. On admission, an acute appendicitis was suspected, founding on arrival blood test Haemoglobin of 11.2 gr/dl, WBC 16.000 cells/microL with Neutrophilia 87%, and prothrombin time of 15%. Once clotting function was corrected by intravenous administration of vitamin K-dependent factors, the patient underwent laparotomy and appendectomy. As intraoperative findings it was observed a gangrenous appendix in subhepatic retrocecal position, surrounded by abundant purulent exudate. Histological study by light microscopy revealed a gangrenous appendix with abscesses and parasitic ova into the submucosal layer of the appendix, suggesting schistosomiasis (see, Figure 1). A macroscopic picture of the specimen removed was not made since it was not suspected this aetiology and no special features were found. Once on word, the patient had an unhurried evolution, developing an adynamic ileus due to hemoperitoneum secondary to anticoagulant treatment. The adynamic ileus was followed by diarrhoea once the ileus was resolved. The patient developed anaemia, with haematocrit decline of 20 points during 7 days without haemodynamic instability, needing blood transfusion, and haematological stabilization after that. As soon as the schistosomiasis diagnosis was done praziquantel 600 mg was administrated orally twice, one-day treatment with 12 h interval divided doses.

Schistosomiasis was described in the first instance by Theodor Bilharz, reported as cause of urinary schistosomiasis in 1851, but it was in 1908 when Pirajá da Silva firstly described the entire disease cycle.

Schistosomes have a typical trematode vertebrate-invertebrate lifecycle, with humans being the definitive host. Cercariae, which are the larvae capable of infecting mammals by enter through the skin host, are highly mobile. The parasite secretes enzymes that break down the skin's protein to enable penetration of the cercarial head through the skin. Once the cercaria penetrates the skin it transforms into a migrating schistosomulum arriving to the lung and days later travel from there to the liver sinusoids. *S. mansoni* and *S. japonicum* worms develop an oral sucker after arriving at the liver, and it is during this period that the parasite begins to feed on red blood cells. Adult worms are about 10 mm long, the longer female worm residing in the gynaecophoric channel of the shorter male, building a paired worm structure (see, Figure 2).

Paired worms of *S. mansoni* and *S. japonicum* migrate from the liver sinusoids to the mesenteric or rectal veins. *S. haematobium* travels from the liver to the perivesical venous plexus of the bladder, urethers, and kidneys through the haemorrhoidal plexus. Once parasites reach maturity they begin to produce eggs, many of the eggs pass through the walls of the blood vessels, and through the intestinal wall, to be passed out of the body in faeces.

Clinically, schistosomiasis is a chronic disease. Acute schistosomiasis, concurrent with eggs spreading throughout blood stream known as Katayama's fever, may occur especially by *S. mansoni* and *S. japonicum*. Clinical manifestations include: abdominal pain and eosinophilia, with cough, diarrhoea, fever, fatigue, and even sudden hepatosplenomegaly.

As chronic disease, continuing infection may cause granulomatous reactions and fibrosis in the affected organs. Clinical manifestations are secondary to the organ involved, and this depends on the affected venous territory by the parasite. In this way, *S. mansoni* and *S. japonicum* damage mainly the organs tributaries to the superior and inferior mesenteric vein which may result in several manifestations as colonic polyposis (*S. mansoni*), portal hypertension with haematemesis and splenomegaly (*S. mansoni*, *S. japonicum*). On the other hand, Cystitis and urethritis with haematuria, which can progress to bladder cancer is produced by *S. haematobium*, because its eggs migrate across the venous plexus of the bladder, urethers and kidneys. Pulmonary hypertension (*S. mansoni*, *S. japonicum*, more rarely *S. haematobium*) [7].

Other clinical manifestations had been reported due to schistosomiasis as acute appendicitis [8–10], cecal mass [11], ovarian tumor [12], or abdominal pain (chronic or acute) concurrent with eosinophilia [13].

Diagnosis of schistosomiasis is governed by the stage of infection. No eggs are generally found in the stools or in the urine during acute schistosomiasis. If no malaria parasites or other apparent cause of fever are detected after travel to tropical and subtropical countries, a history of exposure should be taken. If an infection risk is apparent, a differential blood count is performed, often showing an increased number of eosinophil granulocytes. Finally, serology should be requested to make the diagnosis. This is the usual procedure in suspected cases among tourists, expatriates, and those who visited friends and relatives [1]. Moreover, fellow travellers and family members who have been exposed should also be screened serologically to diagnose asymptomatic infections as stated by Bierman et al. and Schwartz et al. [2, 4]. Migrants with chronic abdominal symptoms should also be asked about possible exposure. Serology and consequently stool and/or urine examinations are performed to detect ova. In his paper Bierman reported a new diagnosis of schistosomiasis in about 26 patients per year in his out-patients clinic, 3% of all new presentations. Infections were almost exclusively acquired in Africa. Eosinophilia was indicative but an insufficient screening tool, and stool and urine microscopy for ova were not sensitive. Screening by serology is easy and reliable and the method of choice in asymptomatic persons with a history of freshwater exposure in a high-risk area [2].

As has been described before, schistosomulum worms develop an oral sucker after arriving at the liver, and it is during this period that the parasite begins to feed on red blood cells. In the case presented the patient suffers anaemia during the postoperative period secondary to haemoperitoneum in an anticoagulant therapy background. But perhaps need be consider an adding factor in the anaemia buildup, the role played by the haemolysis due to the haematophagic behaviour of the parasite.

An essential role is played, in the aetiology of acute appendicitis, by the lumen obstruction of the appendix. Appendiceal lumen obstruction can be founded in several processes; one of them is produced by different parasitic infestation (Helminths as *Enterobius vermicularis*, *Trichuris trichiura*, *Ascaris lumbricoides*, *Strongyloides stercolaris*, *Taenia saginata*, *Schistosoma mansoni*, *Angiostrongylus costaricensis*, and protozoa as *Entamoeba histolytica*, *Balantidium coli*, and *Cryptosporidium parvum*).

Occasionally in the case of schistosomiasis, the histological and parasitological diagnosis cannot guarantee this parasitic infestation as cause of acute appendicitis, mainly when the parasite into the appendiceal lumen cannot be proved. Having said that, particularly in this entity, needs be pointed out that the parasite can produce a heavy haemorrhagic damage once the ova is crossing the full thickness of the vascular and appendiceal wall (See, Figure 3), and in the other hand, the vigorous cellular mediated allergic and inflammatory reaction, induced by the parasite, can produce an important vasculitis on the appendiceal wall. These changes can be enough to trigger the acute appendicitis process without any obstruction of the lumen [14].

Praziquantel is the drug of choice to treat all forms of schistosomiasis. It is active against mature worms. Although resistance to the drug is suspected, the drug can still be used reliably at 40 to 60 mg/kg as a single dose in most cases. Repeated dosages of praziquantel may be necessary in early stages of the disease and to treat long-standing infections. Abdominal discomfort is the most frequently reported side effect of this well-tolerated drug [1].

Finally, should we be aware of schistosomiasis as an underestimated problem in industrialized countries? The three articles by Hatz et al., Bierman et al., and Schwartz et al., and in addition to the reports published by TropNetEurop, raise awareness of the risks and the clinical and diagnostic features of schistosomiasis in industrialized countries [1–4]. The answer is “yes,” it is most probably underreported in industrialized countries, especially among migrants. Because many asymptomatic cases do not progress to severe forms owing to a low parasite load, underreporting may not have serious consequences among tourists and expatriates. Still, when clinical manifestations become apparent and when an unexplained eosinophilia is detected, a proper history of exposure must be assessed and the respective parasitic and serologic tests performed.

As a result of all these considerations and with the changes in global migration, intestinal schistosomiasis should be considered as a cause of acute appendicitis, especially among immigrant patients coming from endemic areas or patients with a history of travel to regions where schistosomiasis is common.

## Figures and Tables

**Figure 1:**
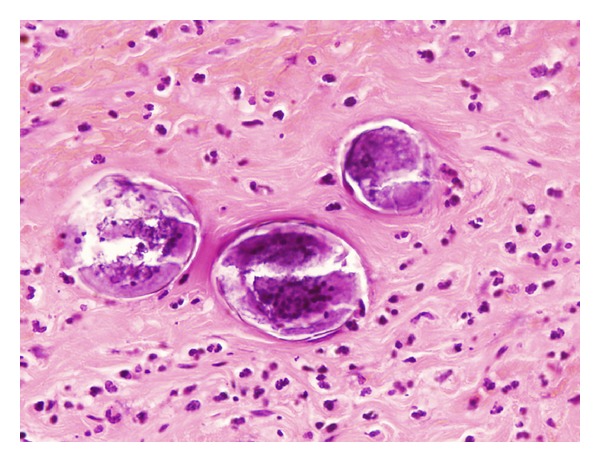
Paraffin embedded 5 *μ*m thick section of appendiceal wall, process from the patient appendix. Magnified image by Light microscopy ×350 and stained with Haematoxylin and Eosin saffron (H-E). The identification of a lateral spine on an oval egg (at the end of an oval egg) is consistent with *S. mansoni*.

**Figure 2:**
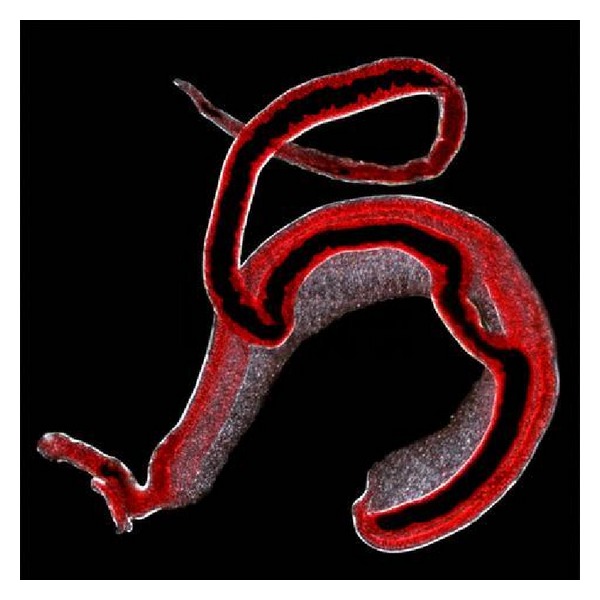
Adult worms paired structure. Magnified image by Light microscopy ×40. can be seen the longer female worm residing in the gynaecophoric channel of the shorter male, building a paired worm structure. From Natural History Museum, London.

**Figure 3:**
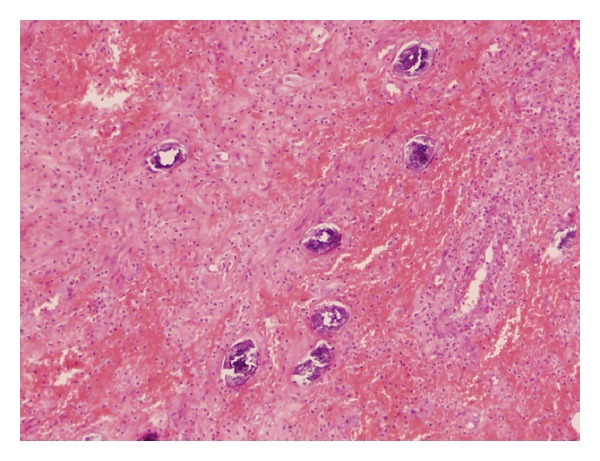
Paraffin embedded 5 *μ*m thick section of appendiceal wall, process from the patient appendix. Magnified image by Light microscopy ×350 and stained with H-E. Can be seen *S. Mansoni* eggs penetrating the appendiceal wall surrounded by heavy haemorraghic infiltrates.
